# P-1794. Evaluating the Impact of a Pharmacy Technician in an Antimicrobial Stewardship Program

**DOI:** 10.1093/ofid/ofae631.1957

**Published:** 2025-01-29

**Authors:** Jennifer Onsrud, Katie Lynn Hammer, Susan Kelly, Lisa Davidson, Tim Pasquale, Susan Bear

**Affiliations:** Atrium Health, charlotte, North Carolina; Atrium Health, charlotte, North Carolina; AtriumHealth, Charlotte, North Carolina; Atrium Health, charlotte, North Carolina; Atrium Health, charlotte, North Carolina; Atrium Health, charlotte, North Carolina

## Abstract

**Background:**

The use of a Certified Pharmacy Technician (CPhT) in clinical roles can optimize and expand pharmacy services. Across our integrated health care system, ceftriaxone is the most utilized inpatient antibiotic. Traditional antibiotic stewardship program (ASP) pharmacists focus on more broad-spectrum agents. To increase review of ceftriaxone, we implemented a novel ASP workflow with a CPhT conducting prospective audit of ceftriaxone.

**Figure:**
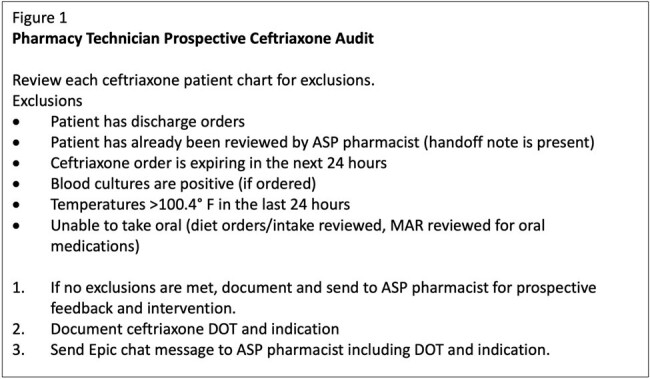

**Methods:**

The CPhT underwent training for ceftriaxone audits, documentation, and communication. Daily chart audits of ceftriaxone were completed with a clinical checklist (Figure 1) and eligible patients were sent to an ASP pharmacist who completed prospective audit and feedback. The primary outcome measure was inpatient administrations of ceftriaxone in days of therapy per 1,000 patient days present (DOT). The secondary outcomes were monthly CPhT audits, audits sent to a pharmacist, related interventions, and total interventions.

Figure 2

**Figure:**
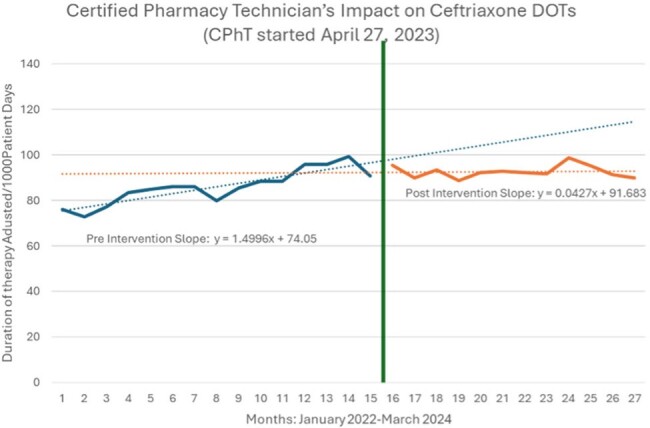

**Results:**

The primary outcome is seen in Figure 2. Although not statistically significant, prior to intervention ceftriaxone use was rising and after intervention, rate of utilization stabilized. Secondary outcomes of monthly CPhT audits, audits sent to a pharmacist, and related interventions can be seen in Figure 3. The monthly average of 452 CPhT audits sent to a pharmacist resulted in 185 related interventions Total interventions in the ASP program during this one-year timeframe were 25,704, which is an 45% increase from the year prior. Using a conservative assumption of 5 mins of pharmacist time avoided per audit by using a CPhT, 62 hours of pharmacist time was saved each month.

Figure 3

**Figure:**
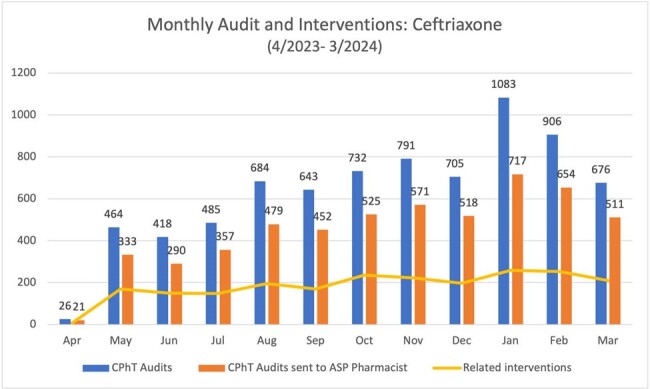

**Conclusion:**

Inclusion of a CPhT into a clinical role in ASP resulted in an increase in prospective audit and feedback of ceftriaxone and total interventions. The use of a CPhT optimized workflow and promoted stewardship of resources. In our experience, a CPhT can be a valuable member of an ASP team and may represent a new clinical role for further consideration. Further evaluation will focus on how this role can be expanded to further optimize stewardship metrics.

Figure 4

**Figure:**
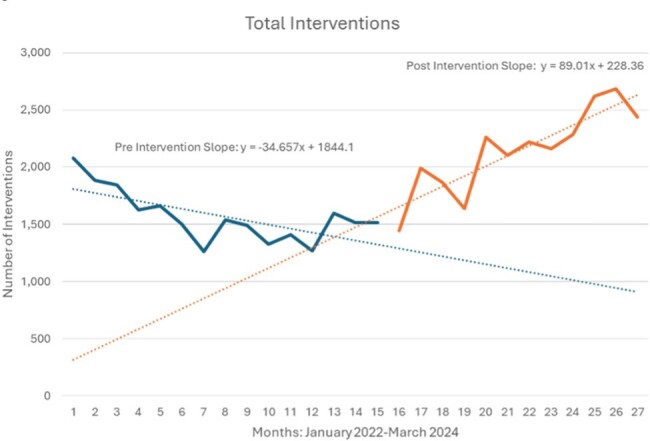

**Disclosures:**

**All Authors**: No reported disclosures

